# Dispersal dynamics of white-tailed deer in human-altered landscapes and implications for disease risk

**DOI:** 10.1371/journal.pone.0325656

**Published:** 2025-06-10

**Authors:** Tayler N. LaSharr, Marie L. J. Gilbertson, Kelsie LaSharr, Michelle Carstensen

**Affiliations:** 1 Haub School of the Environment and Natural Resources, University of Wyoming, 804 E Fremont Street, Laramie, Wyoming, United States of America; 2 Wisconsin Cooperative Wildlife Research Unit, Department of Forest and Wildlife Ecology, University of Wisconsin–Madison, Linden Drive, Madison, Wisconsin, United States of America; 3 Big Game Program, Minnesota Department of Natural Resources, West Broadway, Forest Lake, Minnesota, United States of America; 4 Wildlife Health Program, Minnesota Department of Natural Resources, West Broadway, Forest Lake, Minnesota, United States of America; University of Minnesota, UNITED STATES OF AMERICA

## Abstract

Animal dispersal and migration can play critical roles in population dynamics and species distribution, and these behaviors often are influenced by their environment. The conversion from natural habitats to agricultural lands has altered over 40% of terrestrial surfaces and many wildlife species now inhabit landscapes that are fragmented or heavily composed of agriculture. Understanding how habitat drives patterns of dispersal or migration is of critical importance to population management, particularly in environments that may be changing rapidly with human presence and when considering emerging disease threats. We evaluated how habitat and agriculture in and around white-tailed deer natal ranges in southeastern Minnesota, USA influenced both the probability of dispersal and migration events and the distance animals traveled during those events. Counter to our predictions, we found no evidence that agriculture in the natal range influenced the probability that white-tailed deer would disperse or migrate. Sex, however, played an important role in shaping movement behaviors—compared with resident deer, males were 2.7 times more likely to disperse and 5.2 times less likely to migrate than females. Moreover, while agriculture did not influence the probability of a dispersal event occurring, it did influence the distance traveled with deer dispersing farther in areas with more agriculture and avoiding agriculture during dispersal events. Our results provide insight into the influence of habitat on key movement behaviors that may be extremely important for population management, especially in areas that may have a high prevalence of infectious disease.

## Introduction

Animal dispersal (i.e., movement from a natal range to a permanent, spatially separate range after reaching independence) is an important movement behavior that can underpin population dynamics, influence the distribution of animals across landscapes, and determine fitness [1–3]. Dispersal can minimize inbreeding, improve gene flow, improve access to resources, and reduce competition with conspecifics [4,5], yet dispersal can also present potential disadvantages for both the individual and populations. For individuals, moving into unfamiliar areas can increase their risk of mortality [6] whereas for populations, the influx of new individuals to an area can introduce competition or disease to the existing population [7]. Understanding what influences dispersal in wild animals can provide critical insight into population dynamics and management, especially given that parameters of movement behaviors like dispersal are an important component for many models to understand the spread of mammalian disease [8–10].

Dispersal patterns and the drivers of those patterns can vary dramatically across geographic regions and with shifting climatic conditions or habitats, even within the same species or population [11]. Dispersal events can depend on both the external (e.g., habitat quality or population density) and internal (e.g., age or sex) condition of animals [12,13]. Indeed, habitat on natal ranges can influence both the probability of a dispersal occurring and the distance animals move in ungulate species [14,15]. Given that movement behaviors can largely depend on the environments animals inhabit [16–18] understanding how the environment may influence the likelihood of dispersal events can provide critical information for informed management of wild populations, especially in environments that may be changing rapidly with human presence.

Across the globe, wild populations coexist with human presence, and humans have fundamentally changed the landscape of the earth through infrastructure, recreation, and largely, agriculture. Agriculture and croplands make up over 40% of the world’s terrestrial surfaces, and these large-scale changes in land use have far reaching implications for wild populations [19]. Understanding how animals behave in non-natural landscapes can provide critical insight into population dynamics in these areas, particularly since land use can be fundamental in shaping dispersal events of wild animals [20]. Indeed, red foxes (*Vulpes vulpes*) born into areas with high crop density disperse earlier in life compared with foxes born in areas with fewer crops [21]. Conversely, more fragmented landscapes (i.e., agriculture-natural habitat matrices) limit dispersal events in Meadow brown butterflies (*Maniola jurina* L.; [22]). Yet, other species, like waterfowl, have shifted their habitat use to take advantage of food resources on agricultural land to offset the loss of natural habitats [23]. The use of anthropogenic food resources can alter the fitness of wild animals, from their ability to tolerate pathogens to their ability to survive and raise offspring [24]. Given that drivers of dispersal can vary across both space and time, even within a species, understanding what shapes movement behaviors in human-altered landscapes can be critical to population management.

White-tailed deer (*Odocoileus virginianus*) are habitat generalists and their historic range was characterized by a wide variety of natural habitats from dense deciduous forests and ponderosa pine and conifer forests to semiarid grasslands and desert landscapes [14,25]. With changes to land use in North America over the past two centuries [19], white-tailed deer have become well adapted to living in agricultural landscapes, and these animals exhibit a high degree of heterogeneity in behavior and habitat use both within and between individuals or populations that can have cascading effects on the landscapes they inhabit [26]. Despite their ability to persist in a wide variety of landscapes, their movement behaviors and fitness are still shaped by the environments in which they live [27]. The landscapes deer inhabit can influence home range size [28,29], change the rates and distances animals disperse [14,20,30] and ultimately, determine survival [31]. Indeed, in a mixed forest and agriculture landscape, subadult white-tailed deer in Wisconsin, USA were more likely to disperse during their first spring when they had a high proportion of agriculture in their natal range [30]. Moreover, in a comparative study between two populations, both dispersal and migration of subadult deer were more prevalent in both sexes in a landscape with substantial amounts of agriculture compared with a more natural environment [32]. White-tailed deer therefore present an important system in which to understand drivers of dispersal and dispersal distances, since they occupy a variety of landscapes and often use areas that are heavily composed of agriculture.

Understanding the dispersal ecology of white-tailed deer is an especially important management objective in light of the expanding geographic range and prevalence of chronic wasting disease (CWD) in North America. CWD is a fatal spongiform encephalopathy that can be transmitted rapidly and poses a significant threat to maintaining robust cervid populations [33,34]. The importance of understanding animal movement may be especially important for CWD, because it is transmitted via infectious prions and can be spread through both direct contact and indirect environmental exposure and prions can persist on the landscape for decades [35]. Informed and effective management of CWD requires an understanding of how animals move across time and space, and the factors that shape those movement decisions. The interplay between habitat and disease can be nuanced and complex—habitat hotspots (i.e., habitats that attract large groups) such as breeding sites or foraging areas can increase the spread of infectious disease in wild animals [36]. Understanding how habitat influences movement dynamics can be critical to identifying and managing disease in populations. Here, we provide an understanding of the role landscape characteristics play in the probability of and distance of deer dispersal events.

Using a population of white-tailed deer in southeastern Minnesota, USA that inhabit a mixed agriculture and forested landscape, we sought to understand how agriculture influenced dispersal of a habitat generalist. CWD was discovered in 2016 in this population [37], and understanding drivers of long-distance animal movements presents a pressing management concern. Previous work in this population detected a high number of dispersals by subadults of both sexes (58% male, 21% female; [32]), but the role of habitat characteristics and the environment in shaping these behaviors has not yet been evaluated. To determine how the environment influences dispersal behaviors, and thus, better understand how habitat might ultimately influence disease dynamics, we used a population of white-tailed deer with a recent detection of CWD to evaluate two hypotheses. We hypothesized that habitat and sex would influence the probability of a dispersal occurring (H1) and the distances animals dispersed (H2). Previous work has shown that agriculture can increase both dispersal probabilities and distances [14,30], thus we predicted (P1) deer with a higher proportion of agriculture on their natal range would be more likely to disperse, and (P2) deer with a higher proportion of agriculture along dispersal paths would have longer dispersal distances. Additionally, previous work has demonstrated that dispersals are more common in males than females for many mammals [38,39], thus, to account for the role of sex in influencing dispersal behaviors, we predicted that (P3) male deer would be more likely to disperse than female deer, (P4) male deer would disperse farther than female deer.

In addition, Jennelle et al. also detected migration events in subadults of both sexes (6% male, 20% female) in this population [32]. Similar to dispersal, migration can play an important role in disease dynamics [40] given the potential for CWD prions to contaminate the environment and enhance transmission risk when deer transition between landscapes and occupy additional habitats for extended time periods [41]. Migration patterns can be highly variable between years, depending on factors such as winter severity [42]. While we lack long-term data to disentangle the inter-annual mechanisms underlying migration behaviors, we examined migration behavior in our study where possible to improve our insight into the impact of landscape on long-distance movements relevant to disease transmission and geographic spread.

## Materials and methods

### Study area

**Fig 1:**
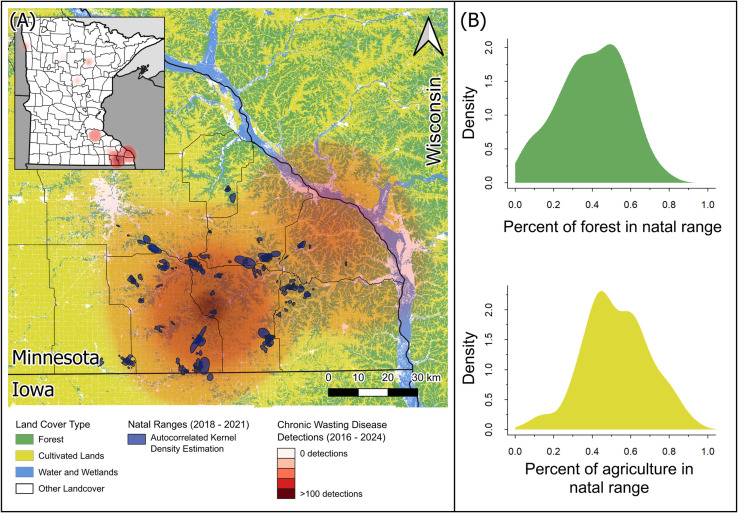
Natal ranges of white-tailed deer, land cover type, and chronic wasting disease detection in southeastern Minnesota, USA. Distribution subadult white-tailed deer in relation to land cover and CWD detections (A) and variation in land cover in natal ranges (B) of subadult white-tailed deer in southeastern Minnesota, USA. Polygons of white-tailed deer represent the autocorrelated kernel density estimations of subadult white-tailed deer prior to their dispersal or migrations (A; 2018–2021). Chronic wasting disease detections (2016–2024) demonstrate this area has the highest prevalence across the state of Minnesota. With the exception of our study system, deer permit areas (thin black boundaries) have had fewer than 20 detections across the state, with most having no detections, since monitoring began in 2016 **(A)**. Land cover in the study area was primarily comprised of agriculture and forest **(A)**, but there was substantial variation among individuals in the proportion of both land cover types in their natal ranges **(B)**. The base layer of this map was derived from the 2019 National Land Cover Database [45].

We evaluated movement of white-tailed deer (hereafter, ‘deer’) in southeastern Minnesota, USA (43.621°, −91.757°) from March 2018 to August 2021. The study area was ~ 7,250 km2 and ownership was primarily private (~90%); habitat was heterogeneous with a mixture of agriculture crops (60%), forest (24%), developed (7%), and the remainder a mix of grasslands, herbaceous wetlands and water (Fig 1; [32,43]). Predominant agricultural crops in the area were corn and soybeans. Common tree species across the study area included sugar maple (*Acer saccharum*), American basswood (*Tilia americana*), bur oak (*Quercus macrocarpa*), northern red oak (*Q. rubra*), white oak (*Q. alba*), swamp white oak (*Q. bicolor*), northern pin oak (*Q. ellisoidalis*), and black oak (*Q. velutina*; [32,44]). Deer in the study area occupied elevations from 180 m to 440 m.

The average modeled density of deer pre-fawning in the deer management unit our study occurred (Deer Management Unit 23) was 30.38 deer/km2 (25–37 deer/km2) during the study period (2018–2021; [46]). The first detection of CWD in free-ranging deer in Minnesota was in 2010; as of 2024, prevalence remains the highest in the southeast corner of the state (Fig 1). During the study period, the study area reflected the highest prevalence for the state (i.e., 1–2% in the study area and <1% elsewhere in the state; [47]). Hunting was the primary source of deer mortality, with harvest ranging from 7.5 to 24.7 deer/km2 [48]. Other predators in the area include coyotes (*Canis latrans*), bobcats (*Lynx rufus*), and domestic dogs (*Canis lupus familiaris*; [32,49]). During the study period, daily temperatures ranged from −35.64°C to 36.35°C, precipitation ranged from 746–1721 mm/year, and there was an average snowfall of 112 cm (National Weather Reporting Station, Preston, MN, USA).

### Animal capture and handling

Each spring (Feb – Mar) from 2018 to 2020, we captured subadult deer (~7–9 months of age) of both sexes by helicopter net gunning using contract capture crews. Capture and handling protocols are described in detail in Jennelle et al. [32]. During periods of potential seasonal dispersal events and during rutting periods, we programmed collars to record locations once every 85 minutes (15 Apr – 15 Jul and 1 Sep – 15 Dec). For the remainder of the year, collars were programmed to collect position data every 3 hours and 45 minutes (~ 6 locations/day).

### Landscape characteristics

An overview of our data collection and analytical workflow can be found in the supplemental materials (S1 Fig). We evaluated the influence of habitat characteristics on dispersal and migratory events using remotely sensed landscape data (S1 Fig). Deer in the study area occupied areas of both southeastern Minnesota and northern Iowa, so we used national landscape databases to ensure consistency across jurisdictions.

To assess the role of land use on range shift behavior, we used land cover data from the National Land Cover Database [45]. Our study area—particularly the area used by deer—is predominantly agricultural and forested, so we followed Gilbertson et al. in focusing on “agricultural” (NLCD codes 81 = pasture/hay; 82 = cultivated crops) or “forested” (41 = deciduous, 42 = evergreen, 43 = mixed forest) land cover [30]. We also considered the impact of waterways on range shift behavior, using the National Hydrography Dataset – Flowlines [50,51]. To focus on reliable or perennial waterways, we excluded intermittent or ephemeral rivers/streams. We also included some waterways classified as “artificial paths”, as this included flowline representations of major water bodies like the Mississippi River. Lower visibility artificial paths (visibility < 250,000) tended to occur as small points on the landscape, rather than distinct flows that might affect deer movement, so these were excluded. We used the waterways linear feature data to generate a 30 meter resolution raster (i.e., the resolution of the other rasters used in our analyses) of distance to the nearest river or stream using the “Euclidean Distance” tool in ArcGIS Pro (v2.8.0). Elevation data was downloaded from the U.S. Geological Survey’s The National Map downloader, as 1 arcsecond resolution digital elevation model (DEM) raster tiles. Individual tiles were then merged into a single raster for the entire study area. All landscape data were processed using the *raster* and *sf* packages in R v4.1.2 [52].

### Delineating movement trajectories

We used previously identified and published dispersal and migratory events for animals in this population (see [32] for detailed methodology). Dispersal events were defined as asymmetrical movements away from a subadult animal’s natal range to a distinct home range occupied in adulthood. Migratory events were defined by seasonal movements between non-overlapping seasonal ranges by both obligate and facultative migrators [14,32,53,54]. We first split movement trajectories into subsets representing five different movement classes: 1) pre-dispersal (movement prior to an observed dispersal event), 2) pre-migration (movement prior to an observed migration event), 3) dispersal, 4) migration, or 5) resident (animals did not shift ranges). Pre-dispersal and resident movements were those between 1 March (spring) or 1 August (autumn) and the date of dispersal (for dispersers) or the median date of dispersal (for residents). Residents were randomly assigned to spring or autumn for analyses so each resident animal only appears in the models once. Pre-migration movements were those from 1 February (spring) or 1 September (autumn) to the date of migration, as migrations often started earlier in the spring and later in the autumn than dispersal events. We required a minimum of 30 GPS (Global Positioning Satellite) fixes and 15 days of monitoring prior to a shift in movement classes (e.g., a shift from pre-dispersal to dispersal). We used 95% vertices from autocorrelated Kernel Density Estimators (aKDEs) to define the distribution of animals on their natal or “pre-shift” range using the *ctmm* package in program R [55]. For each natal range, we calculated the percent forest or agricultural land use based on NLCD classifications within that isopleth. While agriculture makes up a significant part of the study area, deer had substantial variation in the percentage of both forested and agricultural lands in their natal ranges (Fig 1). Individuals with outlier home range areas (greater than three standard deviations larger than the mean) were removed from subsequent analysis.

### Traversability of landscapes

We evaluated the traversability of landscapes (i.e., ease of navigation or movement) by evaluating landscape characteristics in simulated “potential” dispersal paths. We adapted the approach used in [30], where consistent fix rates allowed the use of hidden Markov models (HMM) to parameterize simulated dispersal movements. Here, our fix rates were highly variable, precluding an equivalent HMM approach. Instead, for each observed dispersal event, we fit a gamma distribution to the movement rates (i.e., step length/time between fixes) and a von Mises distribution to the turning angles using the *fitdistrplus* and *circular* packages in R [56,57]. We then calculated the median parameter values across individuals to give a single “movement rate” gamma distribution and “turning angle” von Mises distribution. This approach allows us to directly incorporate empirical data on step lengths and turning angle from the observed dispersal events into the simulated paths. To simulate a single dispersal path, we took 19 random draws from each distribution; across all simulated paths, this produced a median dispersal duration consistent with the observed duration of about 45 hours. Each simulated path started at a random used location in the focal individual’s natal range, and the initial turning angle was selected at random. When simulating subsequent steps in a dispersal path, however, the randomly drawn movement rates needed to be converted to step lengths. Because step length and the time between steps are not fully independent, we fit a linear model for the observed time between fixes as a function of observed movement rates. Then, for each drawn movement rate, we used the fitted linear model to calculate the time between simulated locations and convert the movement rate into a step length.

We simulated 100 potential dispersal paths for each individual. With these paths, we calculated three metrics of traversability: (1) the mean proportion of each simulated path’s steps that were classified as agricultural (mean proportion agricultural); (2) the proportion of simulated paths that intersected a major road (proportion intersecting roads); and (3) the proportion of simulated paths that intersected a river or stream (proportion intersecting rivers/streams).

### Statistical analyses

#### Factors influencing dispersal and migration events.

We evaluated the factors influencing dispersal in subadult deer using logistic regression and if an animal dispersed, migrated, or remained a resident using multinomial regression. We first developed several ‘null’ models to evaluate factors that might influence dispersal or migratory events that were unrelated to our primary hypotheses. For both the logistic regression and multinomial regression models, we evaluated 5 univariate ‘null’ models with 1) home range size 2) number of pre-dispersal locations, 3) span of days before the dispersal or migration event, 4) year, and 5) season as predictor variables. To test for increased probability that dispersal may occur with smaller natal home ranges, we evaluated the influence of home range size (null model 1) on dispersal or migration. To test for potential biases arising from our sampling design we evaluated the number of locations recorded before a movement event (null model 2) and the number of days an animal was monitored before a dispersal event (null model 3). Finally, to test for variability in dispersal that might occur across years or seasons we evaluated the influence of both year (null model 4) and season (null model 5) on probability of dispersal or migratory events occurring. Variables that showed a strong relationship with dispersal probability in null models (natal range size, pre-dispersal fixes, season) were included in full models to account for these effects in our hypothesis testing.

In the full, hypothesis-based logistic regression models to evaluate drivers of dispersal, we used a binary variable of dispersal (0 = non-dispersal, 1 = dispersal) as a response variable. We selected predictor variables based on our hypothesis that sex, land cover, and traversability of landscapes would influence the dispersal of subadult deer. In the models, we included sex, percent agricultural or percent forest cover in the natal range, and the traversability metrics of proportion of simulated paths intersecting rivers/streams, and proportion intersecting roads. We considered additive models, but also models with interactions between season and sex, sex and land cover (agricultural or forest cover in natal range), or season and land cover. We also evaluated a second-order polynomial structure for the number of pre-shift fixes, assuming that very low or high numbers of fixes may be associated with lower dispersal detection (few fixes could result in failure to detect a dispersal event; many fixes could increase the ability to resolve an individual’s use of different, proximate locales into a single range). Model selection was based on Akaike information criterion (AIC), and top models within a ∆AIC of 2 were considered equivalent. We checked for violation of model assumptions using binned residuals [58,59]. Based on initial poor model assumption checks, we excluded the proportion intersecting rivers/streams variable, and included an interaction between home range area and the number of pre-shift fixes.

In the multinomial regression models to evaluate drivers of dispersal, migration, or remaining resident, we included a categorical variable of ‘dispersed’, ‘migrated’, or ‘resident’ as the response variable. Because options for model checking are more limited for multinomial regressions, we fit the top model from the dispersal logistic regression to these data. This model included the percent agricultural land use in the natal range, the proportion intersecting roads, and interactions between sex and season, and home range area and number of pre-shift fixes. Importantly, because no males were observed to migrate in the autumn, we also fit a model with sex and season as simple, additive effects.

#### Factors influencing dispersal distances.

We evaluated the factors influencing how far an animal dispersed using methods developed by Gilbertson et al. [30]. We only evaluated the factors influencing dispersal distances because migrations were limited in number and distance, with limited heterogeneity among hypothesized underlying mechanisms. To identify drivers of dispersal distances, we fit linear regressions with log-transformed range shift distance as the response variable. Dispersal distance was previously determined by Jennelle et al. [32] as the Euclidean distance between GPS fixes at the start and end of range-shift events (as determined by mechanistic range shift analysis; [60]) For dispersers that performed multiple range shifts, we used only the distance between the first and final range in a given dispersal season (i.e., spring or autumn). We developed 5 ‘null’ models following the same protocol developed for analyzing dispersal and migration events.

We evaluated our hypothesis that sex, land cover, and traversability of landscapes would influence the distance that subadult deer dispersed using the following predictor variables: sex, season, proportion intersecting rivers/streams, proportion intersecting roads, and one of three possible land cover variables: percent agricultural cover or forest cover in natal range, or mean proportion agricultural land use in simulated paths (hereafter, proportion agricultural). We considered additive models, but also models with interactions between season and land cover variables, sex and land cover, and nonlinear interactions between season or sex and land cover. We performed model selection using AIC, and model checking was performed with visual assessment of residual and Q-Q plots.

#### Habitat selection during dispersal and migration events.

To evaluate habitat selection during both dispersal and migration events, we fit integrated step selection functions (iSSFs) at both the individual- and the population-level [61–63] across all five movement classes. We resampled trajectories from each of the five movement classes to produce consistent fix rates across individuals, as the scale of fix rates can impact habitat selection inference [64]. The most common fix rates among GPS collared deer were every 0.75, 0.95, 1.25, and 1.95 hours. We therefore resampled for a four hour fix rate with a 30 minute tolerance, which resulted in a median fix rate of 3.76 hours.

We sampled 16 random locations (i.e., steps) for every observed location. We sampled each random step length using independent gamma distributions and each random turning angle using independent von Mises distribution. We extracted habitat characteristics at the end of the step including: agriculture land use (binary variable from NLCD; 1 = agriculture, 0 = non-agriculture), elevation (structured as a second-order polynomial), and distance to nearest river or stream at each random and observed location. Additionally, we evaluated if each random or observed step crossed a road, and included a binary variable of road intersection (1 = intersected road, 0 = did not intersect road). Finally, we included movement variables for step length, log of the step length, and cosine of the turning angle in the models.

For the population-level iSSF models, we fit a single conditional logistic regression model for each movement class using the *TwoStepCLogit* package in R [65]. We included random coefficients for all movement and environmental variables following methods developed by Muff et al. [66] and Gilberston et al. [30]. We scaled and centered elevation and distance to rivers or streams variables at the movement class level. Trajectories without adequate heterogeneity in a covariate will cause both individual- and population-level models to fail to fit; population-level models therefore included only those trajectories for which individual-level models converged. We then compared population-level models across movement classes using log-relative selection strength [67].

For the individual-level iSSF models, we fit an individual model for each trajectory. We scaled and centered the elevation and distance to rivers or streams variables across all trajectories to ensure comparability across models. We then fit an iSSF using a conditional logistic regression for each individual using the *amt* package in R. To understand how selection varied across movement classes, we plotted coefficient estimates across individual-level models. Finally, we fit linear mixed models in a meta-analysis of key predictor variables from the iSSF [64]. We extracted the coefficients for each of the environmental variables from the individual iSSF models and used those coefficients as the response variable in our meta-analysis models. We included movement class, season, and year, as the predictor variables and a random intercept for individual deer.

## Results

We evaluated the factors influencing dispersal and migration events and distances using movement data collected from a total of 142 subadult deer in southeastern Minnesota, USA from 2018 to 2021. The dataset included animals that dispersed (*n* = 41 males and *n* = 17 females), migrated (*n* = 3 males and *n* = 16 females), and remained resident (*n* = 26 males and *n* = 49 females). Sample size for each analysis differed slightly based on the question and the data requirements for analyses (e.g., the analyses evaluating factors influencing dispersal did not include migratory animals); detailed information on sample size is available in the supplementary materials (Table S1).

### Factors influencing dispersal and migration events

**Fig 2:**
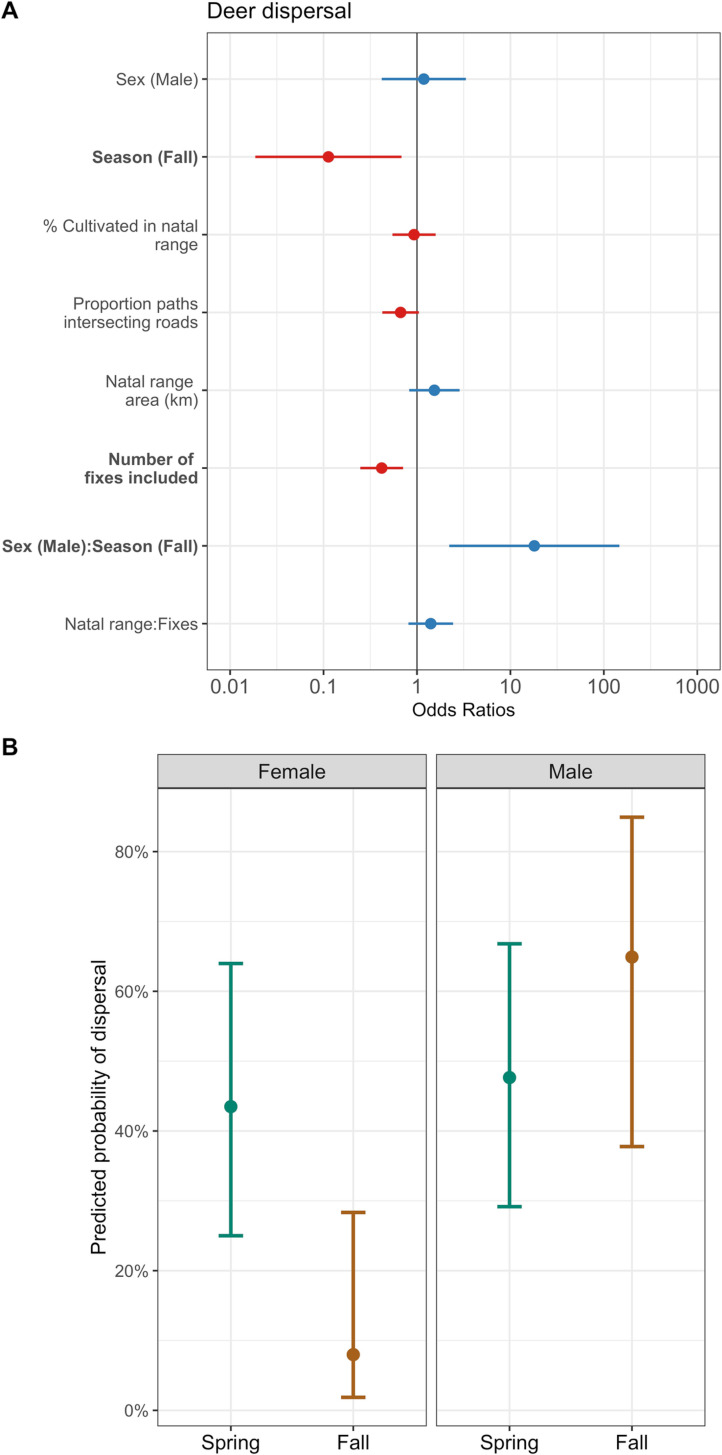
Odds ratios and 95% confidence intervals of covariates influencing the probability of dispersal in subadult white-tailed deer in southeastern Minnesota, USA, from 2018 to 2021. Covariates that increased the odds of dispersal (odds ratios greater than 1) are represented in blue, while covariates that decreased the odds of dispersal (odds ratios less than 1) are represented in red **(A)**. The y-axis is on a logarithmic scale. The predicted effects of sex and season on probability of dispersal **(B)**; female deer were more likely to disperse in the spring (green) compared to the fall (brown).

First, we evaluated the factors influencing if a dispersal event occurred using logistic regression with 124 subadult deer (*n* = 65 females, *n* = 59 males). Our null models indicated that the occurrence or detection of dispersal events was influenced by natal range sizes, the number of locations recorded before the event occurred, and season; we retained all three of these covariates in the final model (Table S2). Our top two models were within Δ2 AIC, with our most parsimonious model indicating that males were more likely to disperse than females (odds ratio = 2.77; 95% CI: 1.16–6.76; Fig 2A; Table S3). When including an interaction between sex and season in our models, we observed some evidence that males were more likely to disperse in the autumn than females (Fig 2B; S2 Fig; S3 Table); given the low sample size of female dispersers—particularly in the autumn—this result should be interpreted with caution. Additionally, neither the percentage of agriculture in an animal’s natal range, the proportion of potential movement paths that intersected a road, nor the size of the natal range influenced probability of dispersal in our top models (Fig 2A; S2 Fig; S3 Table).

Next, we evaluated the factors influencing if an animal dispersed, migrated, or remained resident using multinomial regression with 142 subadult deer (*n* = 78 females, *n* = 64 males). Our null models again indicated that our ability to detect dispersal or migration events were influenced by the size of an animal’s natal (or pre-migration) range, the number of locations recorded before the movement event, and season, thus, we retained all of those covariates in our final multinomial regression model (Table S4; S3 Fig). Our final model indicated that, compared with females, males were 2.74 times more likely to disperse (95% CI: 1.18–6.39; Table 1), and 5.26 times less likely to migrate (odds ratio = 0.19; 95% CI: 0.04–1.03; Table 1). Additionally, the probability of an animal dispersing decreased when the proportion of potential movement paths that intersected a road increased (odds ratio = 0.62; 95% CI: 0.40–0.95; Table 1). Finally, animals were 2.03 times more likely to disperse when they had larger natal ranges (95% CI: 1.14–3.58; Table 1).

**Table 1:** Variables, estimates, standard error (SE), and p-values (P) from a multinomial regression model used to evaluate factors that influence probability of dispersal and migration events of subadult white-tailed deer in southeastern Minnesota, USA from 2018 to 2021.

Behavior	Variable	Exp Est	SE	p-value
Dispersal	Intercept	0.46	0.38	0.04
	Sex (male)	2.74	0.43	0.02
	Season (autumn)	0.78	0.54	0.65
	% Agricultural in pre-shift range	0.99	0.24	0.96
	Proportion paths intersecting roads	0.62	0.22	0.03
	log(Home range area[km])	2.03	0.29	0.02
	Pre-shift fixes	0.46	0.24	<0.01
	log(Home range area[km]) × Pre-shift fixes	1.61	0.29	0.11
Migration	Intercept	0.42	0.45	0.053
	Sex (male)	0.19	0.86	0.050
	Season (autumn)	0.20	1.00	0.10
	% Agricultural in pre-shift range	2.05	0.42	0.08
	Proportion paths intersecting roads	0.77	0.33	0.43
	Log(Home range area[km])	1.75	0.43	0.19
	Pre-shift fixes	0.51	0.34	0.052
	Log(Home range area[km]) × Pre-shift fixes	1.45	0.44	0.40

### Factors influencing dispersal distances

**Fig 3:**
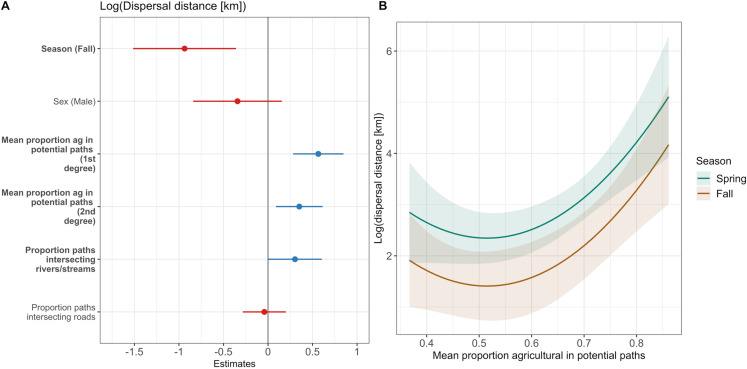
Estimates and 95% confidence intervals of covariates influencing the distance subadult white-tailed deer dispersed in southeastern Minnesota, USA, from 2018 to 2021. Covariates that increased dispersal distances are represented in blue and covariates that decreased dispersal distances are represented in red **(A)**. The predicted effects of season and proportion of agricultural habitat on dispersal distances **(B)**; dispersal distances increased with an increasing proportion of agricultural habitat in potential paths, but the relationship was stronger in spring (green) compared with the autumn (brown).

We evaluated the factors influencing dispersal distances using linear regression with 56 subadult deer (*n* = 17 females, *n* = 39 males). Our null models indicated both home range size and season influenced the distance animals dispersed and we included those covariates in our final model (Table S5). Our top three models were within Δ2 AIC, and our most parsimonious model indicated that animals moved shorter distances in the autumn compared to the spring (*β*  = −0.94; 95% CI: −1.51 – −0.36; Fig 3A; S6 Table). The proportion of paths that intersected rivers and streams positively influenced the distance animals dispersed (*β*  = 0.30; 95% CI: < 0.01–0.60; Fig 3A; S6 Table). Additionally, dispersal distance of subadults increased with a higher amount of agriculture along potential dispersal paths at both the first (*β*  = 0.56; 95% CI: 0.28–0.85; Fig 3A; S6 Table) and second degree (*β*  = 0.35; 95% CI: 0.09–0.61; Fig 3; S4 Fig; S5 Fig; S6 Table). When including an interaction between sex and percent of forest on natal range in our models, at high levels of forest, female dispersal distances increased (S6 Table).

### Habitat selection during dispersal and migration events

We evaluated habitat selection for all five movement classes (dispersal, migration, pre-dispersal, pre-migration, and resident) with both individual- and population-level iSSFs using 142 subadult deer.

**Fig 4:**
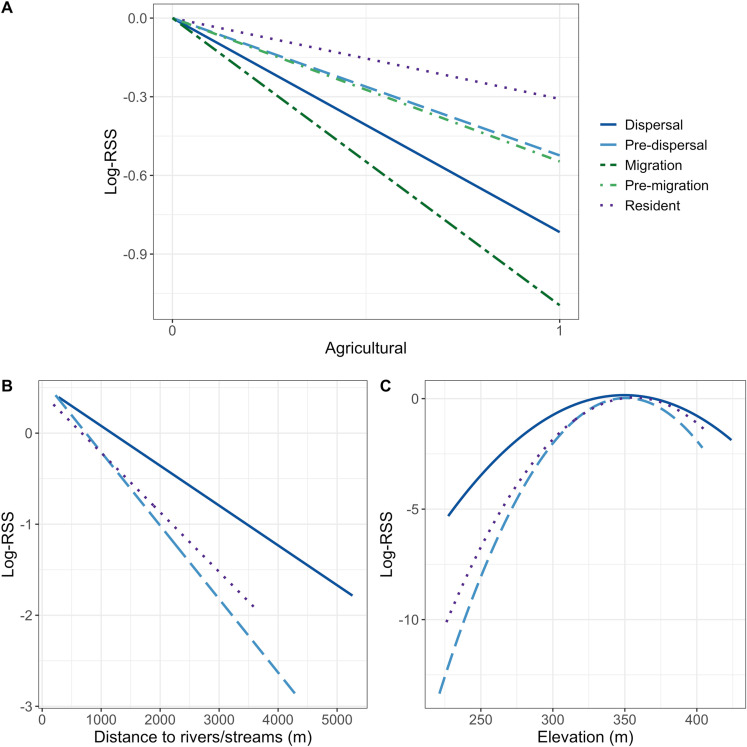
Relative selection strength of white-tailed deer on habitat characteristics. The log-relative strength of selection (log-RSS) for agriculture **(A)**, distance to rivers/streams **(B)**, and elevation (C) by subadult white-tailed deer in southeastern Minnesota, USA from 2018 to 2021 from integrated step selection analyses. Selection was evaluated for five different movement classes: dispersal, pre-dispersal, migration, pre-migration, and resident. Selection coefficients for distance to rivers/streams and elevation were sensitive to the number of random steps for both migration and pre-migration, and thus, are not included in the figure.

The population-level iSSFs revealed that all movement classes demonstrated relative avoidance of agricultural land cover, but avoidance was strongest during dispersal and migration (Table 2; Fig 4A). Resident animals and animals that were still on their natal or pre-shift range (i.e., pre-dispersal and pre-migration) selected areas closer to streams and rivers (Fig 4B) and avoided higher elevations (Fig 4C), but showed no response to either streams and rivers or elevation during dispersal or migration events (Table 2). Migration events were often short, leaving limited data for assessing in a step-selection analysis. As such, migration results are very sensitive to the number of random steps and should be interpreted with caution (S5 Table; S6 Fig). Additionally, the step-selection analysis of dispersal movements were robust to the number of random steps with the exception of distance to rivers/streams. The distance to rivers/streams coefficient estimate was always negative, but the sensitivity of these results supports a cautious non-significant avoidance interpretation. Finally, our meta-analysis of key predictor variables from the individual iSSF models agreed with the population-level models in identifying the strongest relative avoidance for agriculture during dispersal events, but also showed some evidence that this avoidance weakened in the autumn (S7 Table).

**Table 2:** Variables, estimates, standard error (SE), and 95% confidence intervals from integrated step selection analyses for 5 movement classes of subadult white-tailed deer in southeastern Minnesota, USA from 2018 to 2021: 1) dispersal, 2) pre-dispersal, 3) migration, 4) pre-migration, and 5) resident.

Behavior	n	Variable	Estimate	SE	95% CI
Dispersal	14	Step length	0.002	<0.001	(0.002–0.003)
		log(step length)	−0.37	0.09	(−0.55 – −0.20)
		cosine(turning angle)	−0.36	0.13	(−0.61 – −0.11)
		Agricultural land cover	−0.82	0.22	(−1.26 – −0.38)
		Elevation (1st degree)	−0.48	0.69	(−1.83–0.87)
		Elevation (2nd degree)	−0.37	0.29	(−0.93–0.20)
		Distance to rivers/streams	−0.42	0.30	(−1.01–0.18)
Pre-dispersal	55	Step length	<0.001	<0.001	(−0.0002–0.0003)
		log(step length)	0.08	0.03	(0.02–0.13)
		cosine(turning angle)	−0.52	0.04	(−0.61 – −0.44)
		Agricultural land cover	−0.52	0.07	(−0.65 – −0.40)
		Elevation (1st degree)	0.41	0.25	(−0.08–0.91)
		Elevation (2nd degree)	−0.88	0.16	(−1.20 – −0.56)
		Distance to rivers/streams	−0.41	0.13	(−0.66 – −0.16)
Migration	4	Step length	0.003	<0.001	(0.002–0.004)
		log(step length)	−0.35	0.17	(−0.68 – −0.01)
		cosine(turning angle)	−0.54	0.24	(−1.01 – −0.06)
		Agricultural land cover	−1.10	0.49	(−2.05 – −0.14)
		Elevation (1st degree)	−0.59	1.56	(−3.64–2.46)
		Elevation (2nd degree)	−0.41	0.72	(−1.81–1.00)
		Distance to rivers/streams	0.19	0.69	(−1.16–1.53)
Pre-migration	19	Step length	<0.001	<0.001	(−0.0006–0.0004)
		log(step length)	0.05	0.03	(−0.01–0.11)
		cosine(turning angle)	−0.33	0.06	(−0.45 – −0.22)
		Agricultural land cover	−0.55	0.13	(−0.80 – −0.29)
		Elevation (1st degree)	0.62	0.39	(−0.13–1.38)
		Elevation (2nd degree)	−0.57	0.16	(−0.88 – −0.26)
		Distance to rivers/streams	−0.57	0.20	(−0.95 – −0.18)
Resident	74	Step length	<0.001	<0.001	(−0.001 – −0.0005)
		log(step length)	0.22	0.03	(0.16–0.28)
		cosine(turning angle)	−0.46	0.03	(−0.52 – −0.41)
		Agricultural land cover	−0.31	0.07	(−0.44 – −0.18)
		Elevation (1st degree)	0.34	0.29	(−0.22–0.90)
		Elevation (2nd degree)	−1.05	0.18	(−1.41 – −0.69)
		Distance to rivers/streams	−0.58	0.20	(−0.97 – −0.20)

## Discussion

Understanding how different habitat types influence movement behaviors can be paramount to informed management of wildlife populations [16–18], particularly given movement behaviors are a necessary parameter for modeling the spread of infectious disease [8–10]. Habitat and sex were both important drivers of movement behavior in deer in our study, but the influence of habitat differed from expectations in some instances. Contrary to expectations, we found no support for our first hypothesis (H1); habitat did not influence the probability that dispersals or migrations would occur. However, we did find some support for our second hypothesis (i.e., habitat would influence the distances animals traveled during dispersal and migration); aligned with our second prediction (P2), deer did disperse farther distances when there was a high proportion of agriculture along their dispersal paths and exhibited avoidance of agriculture across all movement classes, including dispersal and migration (Fig 3B; Fig 4A; S6 Table). Finally, in support of our final predictions (P3 and P4), sex was an important indicator of whether an animal dispersed or migrated—male deer were more likely to disperse and less likely to migrate than female deer, and male deer dispersed farther distances. Given the heightened concerns for CWD in white-tailed deer in this area (Fig 1), understanding the rate and distances animals will move can help wildlife managers make risk management decisions.

Identifying the role habitat plays in movement behavior can be particularly important when considering disease spread. One of the primary goals for disease management in wild populations is to prevent spread of infectious diseases across the landscape [68] and animal movement can contribute to the transmission of diseases between populations and individuals [69–72]. For cervid populations that are contending with the threat of CWD throughout North America, understanding the drivers of animal movement to different areas through dispersal or migration can be critical for managers to better predict or manage the spread of CWD through time and space—particularly if animals are moving between areas with different disease prevalences. Deer in our study area had some of the highest prevalence for CWD in the state of Minnesota during the study period (i.e., 1–2% in southeast Minnesota and <1% elsewhere in the state; [47]). Given that deer avoided agriculture regardless of their movement class (Fig 4) and moved farther distances with an increasing proportion of agriculture along their dispersal paths (Fig 3), increased agriculture on the landscape may facilitate increased dispersal distances of animals from higher to lower CWD prevalence in Minnesota, USA. Moreover, a lack of significant genetic differentiation across deer populations in this region [73] suggests that geographic barriers are not limiting long-distance movements such as dispersals or migrations; thus, understanding how habitats like agriculture influence long-distance movements is crucial for mitigating and managing disease spread and adjusting surveillance regimes to enhance early detection of new foci.

Counter to expectations, probability of dispersal or migration was not influenced by the habitat in an animal’s natal range. Habitat has been demonstrated to influence dispersal patterns of deer in other populations; in a study conducted in a neighboring state, agriculture on natal ranges increased the probability of dispersal of young deer in Wisconsin, USA [30]. Differences in the composition of habitats between these landscapes may explain the disparity in results between the two studies—the habitat in Wisconsin was more heavily composed of forest (~45%) and less heavily composed of agriculture (~42%) compared with the habitat in our study area (~24% and ~60%, respectively). First, given the proportion of agriculture was high in natal ranges of deer in our study, we may not have had enough variation in deer inhabiting more natural landscapes to detect the influence of agriculture on probability of dispersal. Yet, the behavioral responses by deer to agriculture during dispersal events—avoidance and increased dispersal distances—aligns with previous work [30] and could be driven by both the quality of habitat and available cover deer need to meet their biological needs. Indeed, habitats with heterogeneous cover have been demonstrated to be critical for deer during sensitive periods of the year (e.g., parturition or the mating season; [74,75]) and lack of forested habitats can increase dispersal distance [14]. The primary crops that make up agricultural land in both systems—soybeans and corn—likely provided little or no cover for animals in the spring. Deer in our study traveled 3 - 5x the distance (x̄ = 20 km) that deer in other populations with higher forest cover traveled (x̄ = 4–8.5 km for deer in Georgia, USA, Wisconsin, USA, Pennsylvania, USA, and Illinois, USA; [14,30,32,76,77]). Moreover, while males did disperse more than females as was expected, female dispersal in our study area was much higher compared with other populations (i.e., 21% female in Minnesota, 4% in Wisconsin; [30,32]) and both sexes dispersed farther in the spring compared with the autumn (Fig 3). If the agricultural landscape in our system limits the ability for deer to find suitable habitats to meet their biological needs during a dispersal event, they may need to move farther distances as they seek out desired resources before they settle in a new area.

While the importance of cover for deer may explain the relationship between distances animals moved and the agriculture along their dispersal paths in both sexes, we did find an unexpected relationship in habitat and dispersal distance in females; female deer with an increased proportion of forest cover in their natal ranges moved farther distances while dispersing (S6 Table). These findings were counter to expectations, as forested areas are critical for reproductive females [74], forested landscapes have been demonstrated to reduce dispersal distances [14], and female deer often are highly philopatric and remain close to their natal range (i.e., rose-petal hypothesis; [35,78,79]). The non-linear and unexpected relationship between forest cover and sex on dispersal distance, however, could be due to density dependent processes that result in heavy competition for the limited forest habitat that exists in our study area, especially among females who may require areas with high cover to rear offspring once they become reproductively mature [74]. Indeed, at high densities, females have exhibited increased selection for habitat with more cover during the spring [75] and density estimates of deer in our study were relatively high (i.e., x̄ = 30.38 deer/km^2^) compared with other areas in Minnesota (e.g., ~ 40% of the deer management units had < 10 deer/km^2^ during the study period; [46]). Fine-scale estimates of animal density were not available to include in our analyses; however, accounting for animal density could be an important area of future inquiry given the role density can play on both movement behaviors [54] and disease spread [80]. If density and competition for habitat play an important role in dispersal behaviors of deer, reducing deer density in areas with limited cover could help both reduce disease transmission and geographic spread.

Understanding the interactions between habitat and movement on disease spread can be further complicated by the influence of age-class on both individual behavior and prevalence of CWD in populations. During early stage outbreaks, prevalence of CWD is higher in males compared with females, and most often is found in older age classes [81–84]. Similarly, dispersal events are more common in males than females, but dispersals typically occur during adolescence for mammals [38,39]; indeed while we detected dispersals in both sexes, males were much more likely to disperse than females (Fig 2). During early stage outbreaks, spread of CWD by young, dispersing or migrating animals may be unlikely to occur, but as prevalence in a population increases, young animals can become infected and contribute to disease spread [81]. While prevalence of CWD of animals in our study was high relative to other populations in Minnesota, it was considerably lower than prevalence in other areas throughout white-tailed deer range (i.e., 1–2% in our study area compared with areas in Wisconsin, USA that have prevalence >40% [85]; Fig 1). The likelihood of dispersal by young animals facilitating the spread of CWD to new regions is likely to increase as prevalence increases. Despite the relatively low prevalence in our study area, CWD has been detected in both fawns (~9 months) and yearlings in our study population, demonstrating risk of disease spread through dispersal can still be present at low prevalence [86]. Considering prions can persist in the environment for years and CWD prevalence can change over time, understanding how landscapes and environments influence movement behaviors of young animals in relatively low prevalence areas can help managers prepare for potential spread that may occur if or when prevalence increases and adjust surveillance accordingly.

Given that prevalence of CWD was very low during our study (relative to other populations throughout white-tailed deer range), we did not include any metrics of CWD infection status in our analyses. Previous work has demonstrated that infection status can change movement behaviors in cervids; for example, mule deer (*Odocoileus hemionus*) infected with CWD selected for areas at lower elevations and closer to water sources and moved slower in the months leading up to their mortality [87]. Riparian habitats can potentially contribute to the spread of CWD in cervid populations, especially if animals follow riparian corridors and rivers as they disperse to new areas [88,89] or select areas close to water given polydipsia (i.e., excessive thirst) is a common clinical sign of CWD. While we found no evidence of selection for either elevation or areas close to rivers and streams during dispersal movements, resident deer and animals in their pre-dispersal range did select for higher elevations and areas close to water sources (Fig 3). Considering the prevalence of CWD in our population was relatively low and the animals in our study were young, it is possible that selection for both elevation and water sources along dispersal paths could shift with increasing CWD prevalence if more young animals become infected. Indeed, recent evidence in a population with a relatively high prevalence of CWD (~40%) showed young males exhibited selection for water sources along their dispersal paths [30]. Alternatively, given inferences from iSSFs is dependent on the time between relocations [64], if selection for distance to water sources occurred at a different temporal scale than we used in our analysis, we may not have been able to detect the relationship. Finally, while infection status did not differ between migratory strategies of mule deer—the proportion of migratory animals was similar between infected and non-infected animals [87]—the influence of CWD on dispersal patterns in deer may be more complex. Compared with other North American ungulates, mule deer exhibit high fidelity to migratory strategies once they have reached adulthood [90], whereas drivers of dispersal may be much more dependent on the environments animals inhabit [91–93]. Thus, the role of infection status on dispersal may be more nuanced and future work could evaluate the role of infection status, and the potential interactions between disease, density, and habitat, on dispersal in deer.

Our work provides a preliminary lens in which to evaluate the influence of habitat on movement behavior of young animals in a population that is of heightened disease concern that inhabits an environment heavily composed of agriculture. Several additional factors could be critical drivers of animal movement that were not addressed in these analyses and may be important avenues of future study. First, given the important role density can play on both dispersal probability and disease spread, incorporating fine-scale metrics of density may further elucidate the interactions between habitat, movement, and disease. Additionally, weather conditions can play an important role on long-distance movements in white-tailed deer [42]. Although winters in the study area were relatively mild during the study period [94], winter conditions have been demonstrated to increase the odds of migration by 1.1 per 1-unit increase in deer winter severity index for white-tailed deer [95]. Finally, given that migratory events are facultative for many populations of white-tailed deer [42,53], it is possible that movements defined as dispersal events might actually have been a migration if animals returned to their natal ranges later in life. Future work that monitors individuals for extended periods or for their entire lives could provide more nuanced insight into how habitat influences movement behaviors after animals have reached adulthood. Identifying the underlying drivers of animal movements in wild animals may be critically important when trying to disentangle multiple factors regulating populations, particularly for populations and species that are contending with chronic and fatal diseases.

## Conclusions

Understanding deer movement and habitat selection can inform targeted disease surveillance, control, and prevention measures. Our work provides evidence that even within the same species, the factors underlying migration and dispersal can vary dramatically. We demonstrate that agriculture can play an important role in shaping movement behaviors of white-tailed deer in our system, and surprisingly, water sources were not selected for along migration or dispersal routes. Consequently, predicting spread of CWD based on similar systems in other regions could lead to an inaccurate accounting of risk and resource allocation. To understand drivers of deer movements and the role of habitat in promoting long-distance travel such as dispersal or migration—and consequently, make informed management decisions for populations at risk of disease spread—there is a need for localized understanding of the population.

## Supporting information

S1 Table Sample sizes of subadult white-tailed deer used to evaluate the probability of dispersal, probability of dispersal or migration, the distances animals moved during a dispersal event, and habitat selection using integrated step selection functions (iSSF) in southeastern Minnesota, USA from 2018 to 2021. Parentheses for the iSFF sample sizes denote the sample size pre-dispersal and pre-migration, respectively.
(DOCX)

S2 Table Covariates, estimates, standard error (SE), and p-values (P) from univariate null models to evaluate factors that influence dispersal events unrelated to our primary hypotheses related to dispersal of subadult white-tailed deer in southeastern Minnesota, USA from 2018 to 2021. We evaluated 5 separate models using logistic regression, and included all covariates that were significant in our subsequent analysis: 1) the range area before a dispersal event, 2) the number of fixes before a dispersal event, 3) the number of days an animal was monitored before a dispersal event, 4) the year, and 5) the season.
(DOCX)

S3 Table Estimates, standard error (SE), 95% confidence intervals (CI) and p-value (P) of covariates used to evaluate the factors influencing dispersal events of subadult white-tailed deer in southeastern Minnesota, USA from 2018 to 202 using logistic regression. Both models were within ∆AIC of 2 and were considered equivalent.
(DOCX)

S4 Table Covariates, estimates, standard error (SE), and p-values (P) from univariate null models to evaluate factors that influence dispersal and migration events unrelated to our primary hypotheses related to dispersal of subadult white-tailed deer in southeastern Minnesota, USA from 2018 to 2021. We evaluated 5 separate models using a multinomial regression, and included all covariates that were significant in our subsequent analysis: 1) the range area before a dispersal event, 2) the number of fixes before a dispersal event, 3) the number of days an animal was monitored before a dispersal event, 4) the year, and 5) the season.
(DOCX)

S5 Table Covariates, estimates, standard error (SE), and p-values (P) from univariate null models to evaluate factors that influence dispersal distances unrelated to our primary hypotheses related to dispersal of subadult white-tailed deer in southeastern Minnesota, USA from 2018 to 2021. We evaluated 5 separate models using linear regression, and included all covariates that were significant in our subsequent analysis: 1) the range area before a dispersal event, 2) the number of fixes before a dispersal event, 3) the number of days an animal was monitored before a dispersal event, 4) the year, and 5) the season.
(DOCX)

S6 Table Estimates, standard error (SE), 95% confidence intervals (CI) and p-value (P) of covariates used to evaluate the factors influencing dispersal distances of subadult white-tailed deer in southeastern Minnesota, USA from 2018 to 2021 using linear regression. All models were within ∆AIC of 2 and were considered equivalent.
(DOCX)

S7 Table Variables, estimates, standard error (SE), and p-value (P) from a meta-analyses to determine factors influencing selection for agricultural land use from the integrated step selection function of subadult white-tailed deer in southeastern Minnesota, USA from 2018 to 2021. (DOCX)

S1 Fig Conceptual diagram of the analytical workflow used to identify the role of habitat on probability of dispersal and migration, dispersal distances, and habitat selection during dispersal events of subadult white-tailed deer in southeastern Minnesota, USA, from 2018 to 2021. Each box represents a different stage of the workflow, with data preparation in blue and statistical models in purple. Covariate prep included classifying agriculture and forested land cover types form the National Land Cover Database (NLCD) and creating a raster of distance to water using the National Hydrography Dataset (NHD). The statistical models used for each of the analyses are indicated parenthetically in their respective boxes.
(TIF)

S2 Fig Odds ratios and 95% confidence intervals of covariates influencing the probability of dispersal in subadult white-tailed deer in southeastern Minnesota, USA, from 2018 to 2021 from the second model within 2 AIC of the top model reported in the manuscript (Table S3). Covariates that increased the odds of dispersal (odds ratios greater than 1) are represented in blue, while covariates that decreased the odds of dispersal (odds ratios less than 1) are represented in red (A). The y-axis is on a logarithmic scale.
(TIF)

S3 Fig Predicted probability of subadult white-tailed deer in southeastern Minnesota remaining resident, dispersing, or migrating based on the proportion of paths that intersected major roads from 2018 to 2021. (TIF)

S4 Fig Estimates and 95% confidence intervals of covariates influencing the distance subadult white-tailed deer dispersed in southeastern Minnesota, USA, from 2018 to 2021. Covariates that increased dispersal distances are represented in blue and covariates that decreased dispersal distances are represented in red (A). Distance dispersed increased with an increasing proportion of agricultural habitat in potential paths (B), but the relationship was dependent on sex, with females exhibiting a nonlinear relationship with dispersal distances lowest at average proportion of agriculture.
(TIF)

S5 Fig Estimates and 95% confidence intervals of covariates influencing the distance subadult white-tailed deer dispersed in southeastern Minnesota, USA, from 2018 to 2021. Covariates that increased dispersal distances are represented in blue and covariates that decreased dispersal distances are represented in red (A). Distance dispersed changes with an increasing proportion of agricultural habitat in potential paths (B), but the relationship was dependent on sex; males (green) had a linear decline in distance traveled with higher proportion of forest in their natal range, whereas females (purple) exhibited a nonlinear relationship with highest dispersal distances at low and high forest cover in natal ranges.
(TIF)

S6 Fig Coefficient estimates from integrated step selection functions with an increasing number of random steps for dispersal, pre-dispersal, migration, pre-migration, and non-dispersal movements. The black dashed lined represents the number of random steps used in the final analyses (16 steps). Estimates for all covariates during migration were very sensitive to the number of steps included. Estimates for elevation during pre-migration were sensitive the number of steps included. Estimates for distance to rivers during dispersal were also sensitive to the number of steps included. Analyses were conducted on subadult white-tailed deer dispersed in southeastern Minnesota, USA, from 2018 to 2021.
(TIF)

S7 Fig Selection for agriculture by subadult white-tailed deer dispersed in southeastern Minnesota, USA, from 2018 to 2021 in spring and autumn for five different movement classes: dispersal (dark blue), pre-dispersal (light blue), migration (dark green), pre-migration (light green), and resident (purple). Box and whisker plots represent the full range of data, with the upper and lower quantile defined by the box, and the median value by the middle line. Animals avoided agriculture across all five movement classes in spring. Sample size was limited in autumn for all classes expect pre-dispersal and resident, and should be interpreted with caution.
(TIF)
