# Supplementary material for: Dispersal dynamics of white-tailed deer in human-altered landscapes and implications for disease risk
Source: PLoS One. 2025 Jun 10;20(6):e0325656. doi: 10.1371/journal.pone.0325656 (PMC12151444; doi:10.1371/journal.pone.0325656)
Supplement: S1 Table — Parentheses for the iSFF sample sizes denote the sample size pre-dispersal and pre-migration, respectively. (DOCX) [file pone.0325656.s001.docx]

Table S1. Sample sizes of juvenile white-tailed deer used to evaluate the probability of dispersal, probability of dispersal or migration, the distances animals moved during a dispersal event, and habitat selection using integrated step selection functions (iSSF) in southeastern Minnesota, USA from 2018 to 2021. Parentheses for the iSFF sample sizes denote the sample size pre-dispersal and pre-migration, respectively.

| **Analysis** |  | **Dispersers** | **Migrators** | **Residents** |
| --- | --- | --- | --- | --- |
| Dispersal logistic regression | Total | 51 | 0 | 73 |
|  | Spring | 36 | 0 | 36 |
|  | Fall | 15 | 0 | 37 |
|  | Male | 35 | 0 | 24 |
|  | Female | 16 | 0 | 49 |
| Range shift multinomial regression | Total | 55 | 14 | 73 |
|  | Spring | 40 | 12 | 36 |
|  | Fall | 15 | 2 | 37 |
|  | Male | 38 | 2 | 24 |
|  | Female | 17 | 12 | 49 |
| Range shift distance linear regression | Total | 56 | 14 | 0 |
|  | Spring | 41 | 12 | 0 |
|  | Fall | 15 | 2 | 0 |
|  | Male | 39 | 2 | 0 |
|  | Female | 17 | 12 | 0 |
| Individual-level iSSF | Total | 29 (58) | 5 (19) | 75 |
|  | Spring | 23 (43) | 5 (17) | 37 |
|  | Fall | 6 (15) | 0 (2) | 38 |
|  | Male | 18 (41) | 2 (3) | 26 |
|  | Female | 11 (17) | 3 (16) | 49 |
