# Supplementary material for: Dispersal dynamics of white-tailed deer in human-altered landscapes and implications for disease risk
Source: PLoS One. 2025 Jun 10;20(6):e0325656. doi: 10.1371/journal.pone.0325656 (PMC12151444; doi:10.1371/journal.pone.0325656)
Supplement: S3 Table — Both models were within ∆AIC of 2 and were considered equivalent. (DOCX) [file pone.0325656.s003.docx]

Table S3. Estimates, standard error (SE), 95% confidence intervals (CI) and p-value (P) of covariates used to evaluate the factors influencing dispersal events of juvenile white-tailed deer in southeastern Minnesota, USA from 2018 to 202 using logistic regression. Both models were within ∆AIC of 2 and were considered equivalent.

| Model | Variable | Exp(Est) | SE | 95% CI | P |
| --- | --- | --- | --- | --- | --- |
| 1 | Intercept | 0.47 | 0.40 | (0.21 – 1.01) | 0.06 |
|  | Sex (male) | 2.77 | 0.45 | (1.16 – 6.76) | 0.02 |
|  | Season (autumn) | 0.61 | 0.58 | (0.19 – 1.86) | 0.39 |
|  | % Agriculture in natal range | 1.00 | 0.25 | (0.61 – 1.64) | 1.00 |
|  | Proportion paths intersecting roads | 0.66 | 0.22 | (0.42 – 1.02) | 0.06 |
|  | Log(home range area[km]) | 1.65 | 0.31 | (0.92 – 3.11) | 0.10 |
|  | Pre-shift fixes | 0.41 | 0.27 | (0.23 – 0.67) | <0.001 |
|  | Home range area: pre-shift fixes | 1.45 | 0.28 | (0.85 – 2.58) | 0.18 |
| 2 | Intercept | 0.77 | 0.43 | (0.33 – 1.77) | 0.54 |
|  | Sex (male) | 1.18 | 0.53 | (0.42 – 3.36) | 0.75 |
|  | Season (autumn) | 0.11 | 0.92 | (0.01 – 0.60) | 0.02 |
|  | % Agriculture in natal range | 0.93 | 0.27 | (0.54 – 1.59) | 0.79 |
|  | Proportion paths intersecting roads | 0.67 | 0.23 | (0.42 – 1.04) | 0.08 |
|  | Log(home range area[km]) | 1.54 | 0.32 | (0.83 – 2.93) | 0.18 |
|  | Pre-shift fixes | 0.42 | 0.27 | (0.24 – 0.69) | <0.01 |
|  | Sex (male): Season (autumn) | 18.03 | 1.07 | (2.49 – 182.75) | <0.01 |
|  | Home range area: pre-shift fixes | 1.40 | 0.28 | (0.81 – 2.49) | 0.23 |
