# Supplementary material for: Dispersal dynamics of white-tailed deer in human-altered landscapes and implications for disease risk
Source: PLoS One. 2025 Jun 10;20(6):e0325656. doi: 10.1371/journal.pone.0325656 (PMC12151444; doi:10.1371/journal.pone.0325656)
Supplement: S4 Table — We evaluated 5 separate models using a multinomial regression, and included all covariates that were significant in our subsequent analysis: 1) the range area before a dispersal event, 2) the number of fixes before a dispersal event, 3) the number of days an animal was monitored before a dispersal event, 4) the year, and 5) the season. (DOCX) [file pone.0325656.s004.docx]

Table S4. Covariates, estimates, standard error (SE), and p-values (P) from univariate null models to evaluate factors that influence dispersal and migration events unrelated to our primary hypotheses related to dispersal of juvenile white-tailed deer in southeastern Minnesota, USA from 2018 to 2021. We evaluated 5 separate models using a multinomial regression, and included all covariates that were significant in our subsequent analysis: 1) the range area before a dispersal event, 2) the number of fixes before a dispersal event, 3) the number of days an animal was monitored before a dispersal event, 4) the year, and 5) the season.

| Model | Covariate | Behavior | Estimate | SE | P |
| --- | --- | --- | --- | --- | --- |
| 1 | Pre-dispersal range area (km) | Dispersal | 0.861 | 0.220 | 0.000 |
|  |  | Migration | 0.829 | 0.321 | 0.010 |
| 2 | Pre-dispersal fixes | Dispersal | -0.541 | 0.196 | 0.006 |
|  |  | Migration | -0.580 | 0.307 | 0.059 |
| 3 | Days monitored prior to dispersal | Dispersal | -0.199 | 0.185 | 0.281 |
|  |  | Migration | -0.464 | 0.277 | 0.094 |
| 4 | Year (2019) | Dispersal | 0.808 | 0.428 | 0.059 |
|  |  | Migration | 0.882 | 0.689 | 0.200 |
|  | Year (2020) | Dispersal | 0.024 | 0.466 | 0.959 |
|  |  | Migration | -0.056 | 0.813 | 0.945 |
| 5 | Season (autumn) | Dispersal | -1.008 | 0.383 | 0.008 |
|  |  | Migration | -1.819 | 0.799 | 0.023 |
