# Supplementary material for: Dispersal dynamics of white-tailed deer in human-altered landscapes and implications for disease risk
Source: PLoS One. 2025 Jun 10;20(6):e0325656. doi: 10.1371/journal.pone.0325656 (PMC12151444; doi:10.1371/journal.pone.0325656)
Supplement: S5 Table — We evaluated 5 separate models using linear regression, and included all covariates that were significant in our subsequent analysis: 1) the range area before a dispersal event, 2) the number of fixes before a dispersal event, 3) the number of days an animal was monitored before a dispersal event, 4) the year, and 5) the season. (DOCX) [file pone.0325656.s005.docx]

Table S5. Covariates, estimates, standard error (SE), and p-values (P) from univariate null models to evaluate factors that influence dispersal distances unrelated to our primary hypotheses related to dispersal of juvenile white-tailed deer in southeastern Minnesota, USA from 2018 to 2021. We evaluated 5 separate models using linear regression, and included all covariates that were significant in our subsequent analysis: 1) the range area before a dispersal event, 2) the number of fixes before a dispersal event, 3) the number of days an animal was monitored before a dispersal event, 4) the year, and 5) the season.

| Model | Covariate | Estimate | SE | P |
| --- | --- | --- | --- | --- |
| 1 | Pre-dispersal range area (km) | 0.240 | 0.110 | 0.032 |
| 2 | Pre-dispersal fixes | 0.101 | 0.113 | 0.376 |
| 3 | Days monitored prior to dispersal | 0.070 | 0.113 | 0.537 |
| 4 | Year (2019) | 0.269 | 0.265 | 0.313 |
|  | Year (2020) | 0.130 | 0.307 | 0.674 |
| 5 | Season (autumn) | -0.723 | 0.248 | 0.005 |
