# Supplementary material for: Dispersal dynamics of white-tailed deer in human-altered landscapes and implications for disease risk
Source: PLoS One. 2025 Jun 10;20(6):e0325656. doi: 10.1371/journal.pone.0325656 (PMC12151444; doi:10.1371/journal.pone.0325656)
Supplement: S6 Table — All models were within ∆AIC of 2 and were considered equivalent. (DOCX) [file pone.0325656.s006.docx]

Table S6. Estimates, standard error (SE), 95% confidence intervals (CI) and p-value (P) of covariates used to evaluate the factors influencing dispersal distances of juvenile white-tailed deer in southeastern Minnesota, USA from 2018 to 2021 using linear regression. All models were within ∆AIC of 2 and were considered equivalent.

| Model | Variable | Est | SE | 95% CI | P |
| --- | --- | --- | --- | --- | --- |
| 1 | Intercept | 2.57 | 0.23 | (2.12 – 3.03) | <0.001 |
|  | Season (autumn) | -0.94 | 0.29 | (-1.51 – -0.36) | <0.01 |
|  | Sex (male) | -0.34 | 0.25 | (-0.84 – 0.15) | 0.17 |
|  | Mean proportion agriculture in paths (1st degree) | 0.56 | 0.14 | (0.28 – 0.85) | <0.001 |
|  | Mean proportion agriculture in paths (2nd degree) | 0.35 | 0.13 | (0.09 – 0.61) | <0.01 |
|  | Proportion paths intersecting rivers/streams | 0.30 | 0.15 | (0.00 – 0.60) | 0.05 |
|  | Proportion paths intersecting roads | -0.04 | 0.12 | (-0.28 – 0.20) | 0.73 |
| 2 | Intercept | 2.37 | 0.26 | (1.86 – 2.89) | <0.001 |
|  | Season (autumn) | -1.06 | 0.30 | (-1.65 – -0.46) | <0.001 |
|  | Sex (male) | 0.00 | 0.33 | (-0.66 – 0.66) | 1.00 |
|  | Mean proportion agriculture in paths (1st degree) | 0.46 | 0.24 | (-0.03 – 0.95) | 0.06 |
|  | Mean proportion agriculture in paths (2nd degree) | 0.69 | 0.25 | (0.19 – 1.19) | <0.01 |
|  | Proportion paths intersecting rivers/streams | 0.25 | 0.16 | (-0.07 – 0.57) | 0.13 |
|  | Proportion paths intersecting roads | -0.06 | 0.12 | (-0.30 – 0.18) | 0.64 |
|  | Male: agriculture in paths (1st degree) | 0.07 | 0.28 | (-0.49 – 0.63) | 0.81 |
|  | Male: agriculture in paths (2nd degree) | -0.44 | 0.28 | (-1.01 – 0.13) | 0.13 |
| 3 | Intercept | 2.46 | 0.26 | (1.95 – 2.97) | <0.001 |
|  | Season (autumn) | -1.07 | 0.28 | (-1.64 – -0.50) | <0.001 |
|  | Sex (male) | 0.13 | 0.33 | (-0.53 – 0.79) | 0.69 |
|  | % Forest in natal range (1st degree) | -0.20 | 0.21 | (-0.63 – 0.23) | 0.34 |
|  | % Forest in natal range (2nd degree) | 0.50 | 0.18 | (0.13 – 0.87) | <0.01 |
|  | Proportion paths intersecting rivers/streams | 0.16 | 0.14 | (-0.13 – 0.45) | 0.26 |
|  | Proportion paths intersecting roads | -0.11 | 0.12 | (-0.35 – 0.13) | 0.38 |
|  | Male:% forest (1st degree) | -0.30 | 0.26 | (-0.82 – 0.22) | 0.25 |
|  | Male:% forest (2nd degree) | -0.47 | 0.21 | (-0.90 – -0.04) | 0.03 |
