# Supplementary material for: Dispersal dynamics of white-tailed deer in human-altered landscapes and implications for disease risk
Source: PLoS One. 2025 Jun 10;20(6):e0325656. doi: 10.1371/journal.pone.0325656 (PMC12151444; doi:10.1371/journal.pone.0325656)
Supplement: S7 Table — (DOCX) [file pone.0325656.s007.docx]

Table S7. Variables, estimates, standard error (SE), and p-value (P) from a meta-analyses to determine factors influencing selection for agricultural land use from the integrated step selection function of juvenile white-tailed deer in southeastern Minnesota, USA from 2018 to 2021.

| Model | Variable | Estimate | Std. Error | P |
| --- | --- | --- | --- | --- |
| Agricultural land use | Intercept | -0.57 | 0.13 | <0.001 |
|  | Movement class: pre-dispersal | -0.09 | 0.14 | 0.52 |
|  | Movement class: dispersal | -0.65 | 0.22 | <0.01 |
|  | Season: autumn | 0.43 | 0.14 | <0.01 |
|  | Year: 2019 | 0.13 | 0.16 | 0.41 |
|  | Year: 2020 | 0.06 | 0.16 | 0.74 |
