# Supplementary figures and images for: Dispersal dynamics of white-tailed deer in human-altered landscapes and implications for disease risk
Source: PLoS One. 2025 Jun 10;20(6):e0325656. doi: 10.1371/journal.pone.0325656 (PMC12151444; doi:10.1371/journal.pone.0325656)

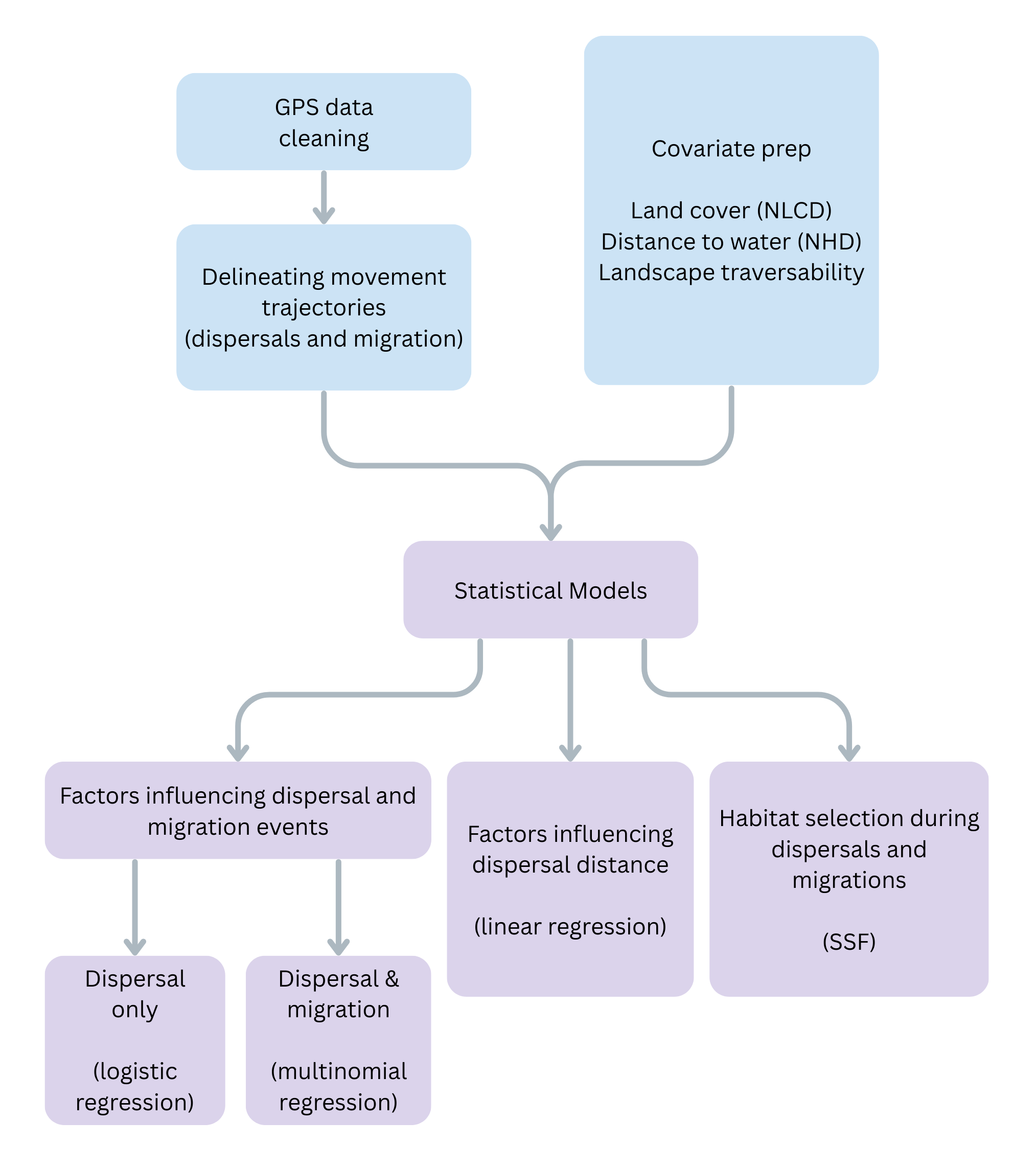

Supplement: S1 Fig — Each box represents a different stage of the workflow, with data preparation in blue and statistical models in purple. Covariate prep included classifying agriculture and forested land cover types form the National Land Cover Database (NLCD) and creating a raster of distance to water using the National Hydrography Dataset (NHD). The statistical models used for each of the analyses are indicated parenthetically in their respective boxes. (TIF) [file pone.0325656.s008.tif]

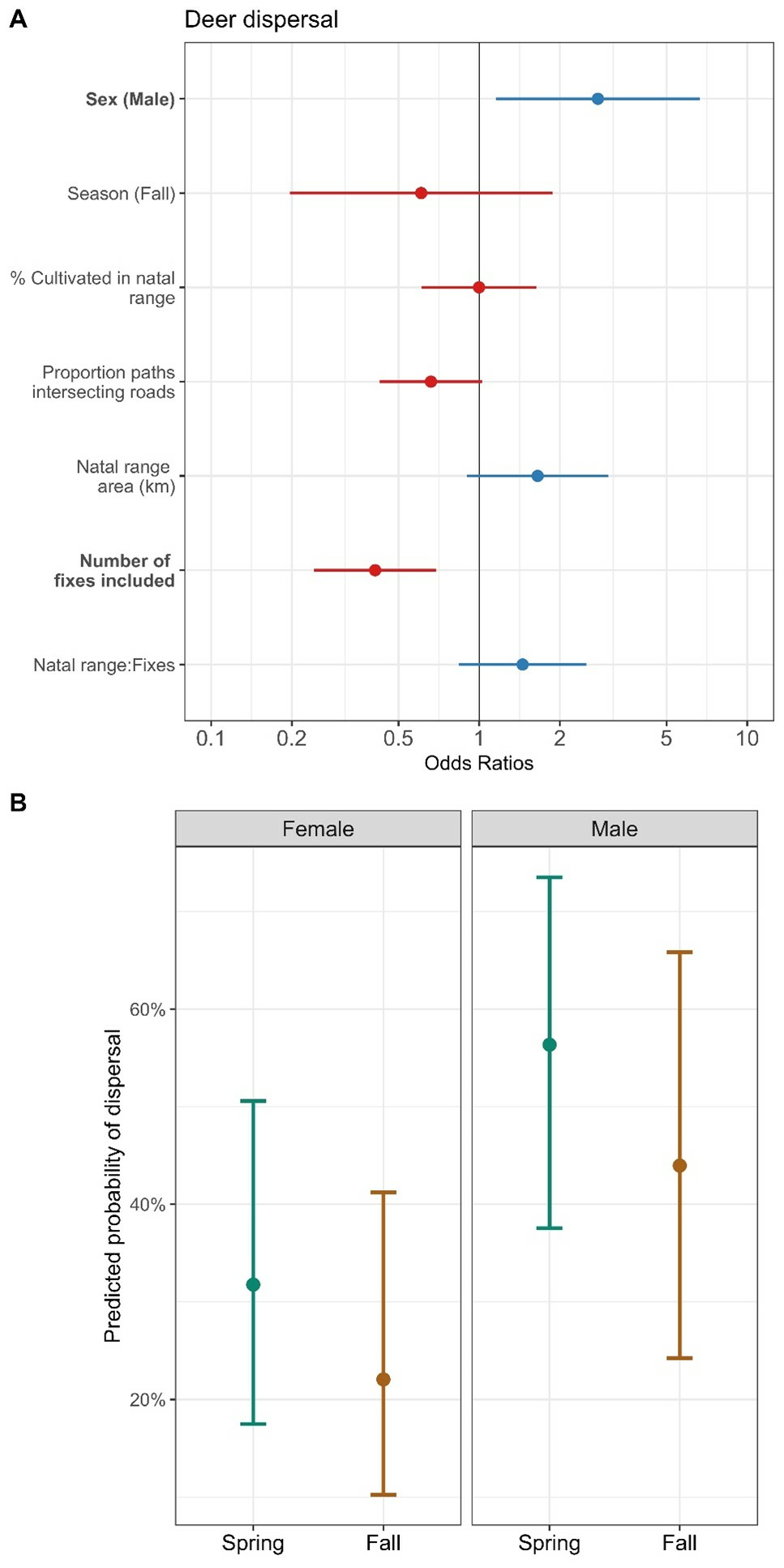

Supplement: S2 Fig — Covariates that increased the odds of dispersal (odds ratios greater than 1) are represented in blue, while covariates that decreased the odds of dispersal (odds ratios less than 1) are represented in red (A). The y-axis is on a logarithmic scale. (TIF) [file pone.0325656.s009.tif]

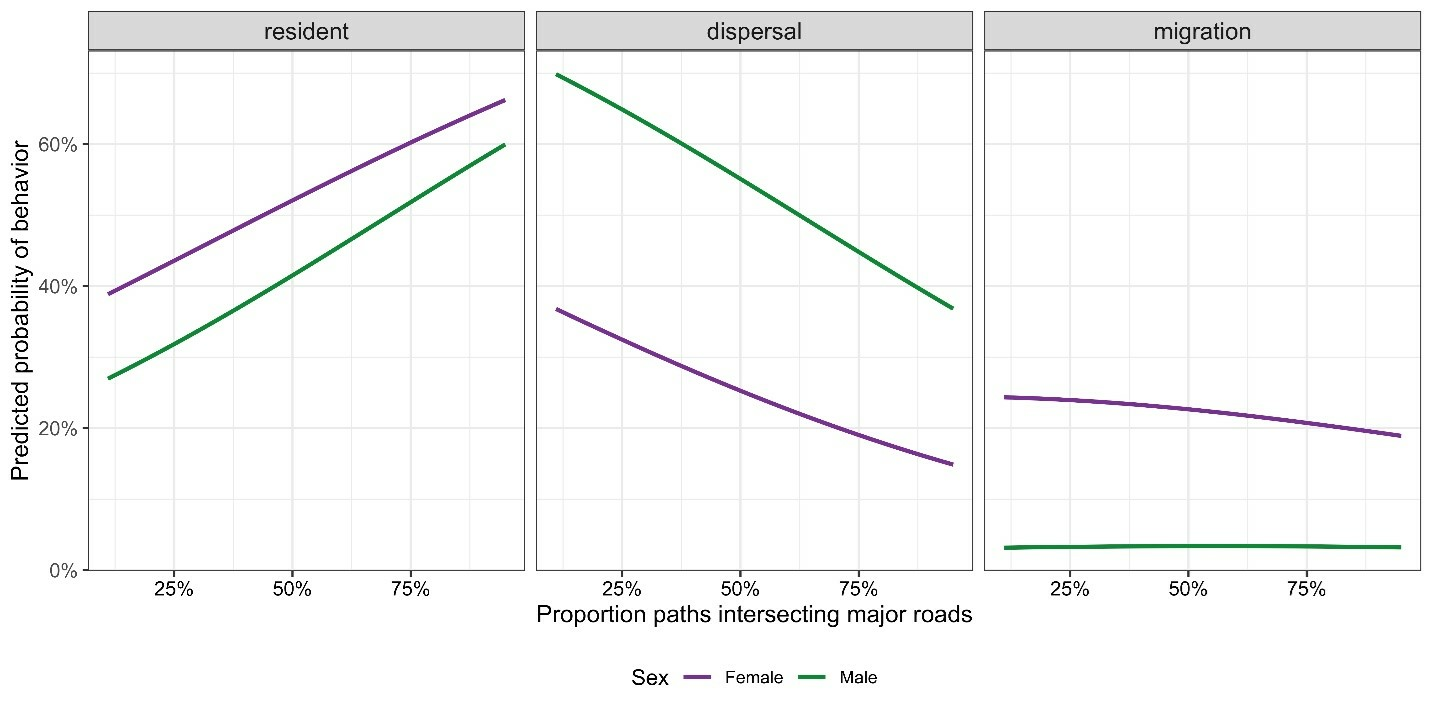

Supplement: S3 Fig — (TIF) [file pone.0325656.s010.tif]

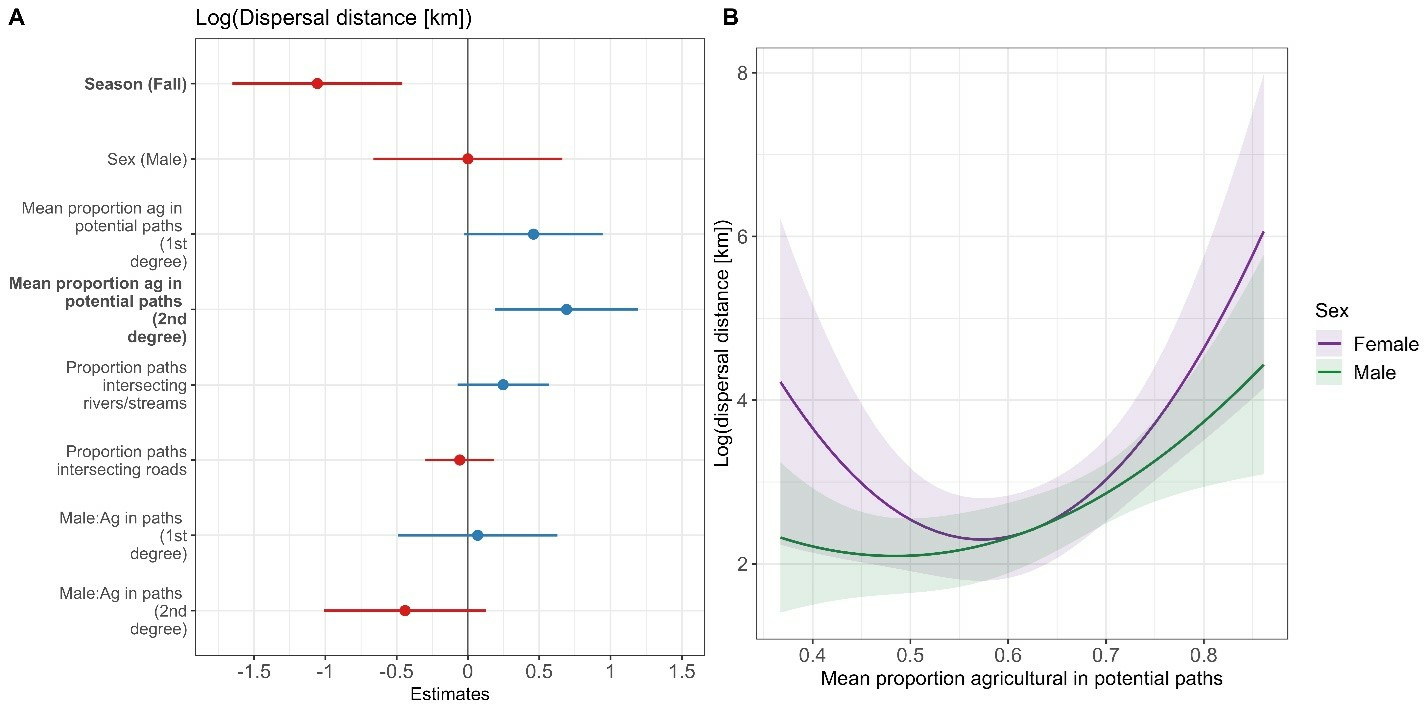

Supplement: S4 Fig — Covariates that increased dispersal distances are represented in blue and covariates that decreased dispersal distances are represented in red (A). Distance dispersed increased with an increasing proportion of agricultural habitat in potential paths (B), but the relationship was dependent on sex, with females exhibiting a nonlinear relationship with dispersal distances lowest at average proportion of agriculture. (TIF) [file pone.0325656.s011.tif]

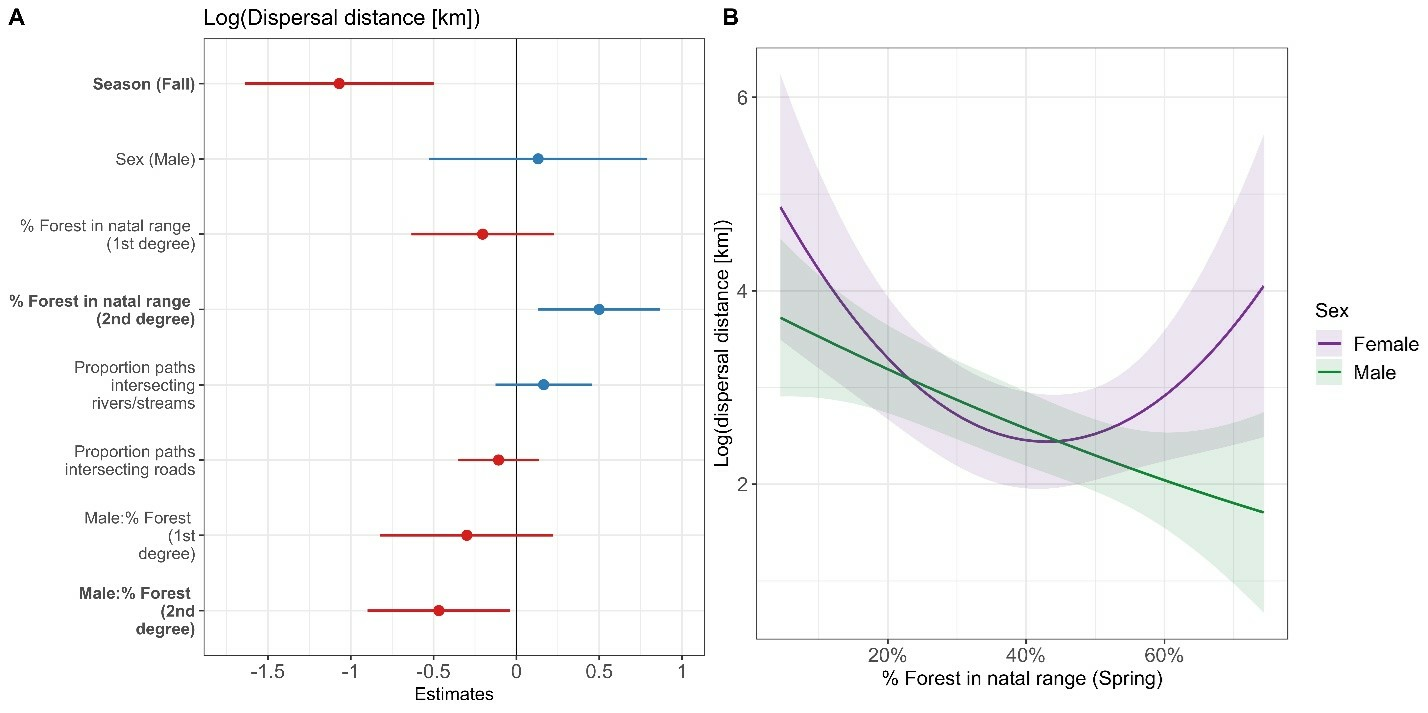

Supplement: S5 Fig — Covariates that increased dispersal distances are represented in blue and covariates that decreased dispersal distances are represented in red (A). Distance dispersed changes with an increasing proportion of agricultural habitat in potential paths (B), but the relationship was dependent on sex; males (green) had a linear decline in distance traveled with higher proportion of forest in their natal range, whereas females (purple) exhibited a nonlinear relationship with highest dispersal distances at low and high forest cover in natal ranges. (TIF) [file pone.0325656.s012.tif]

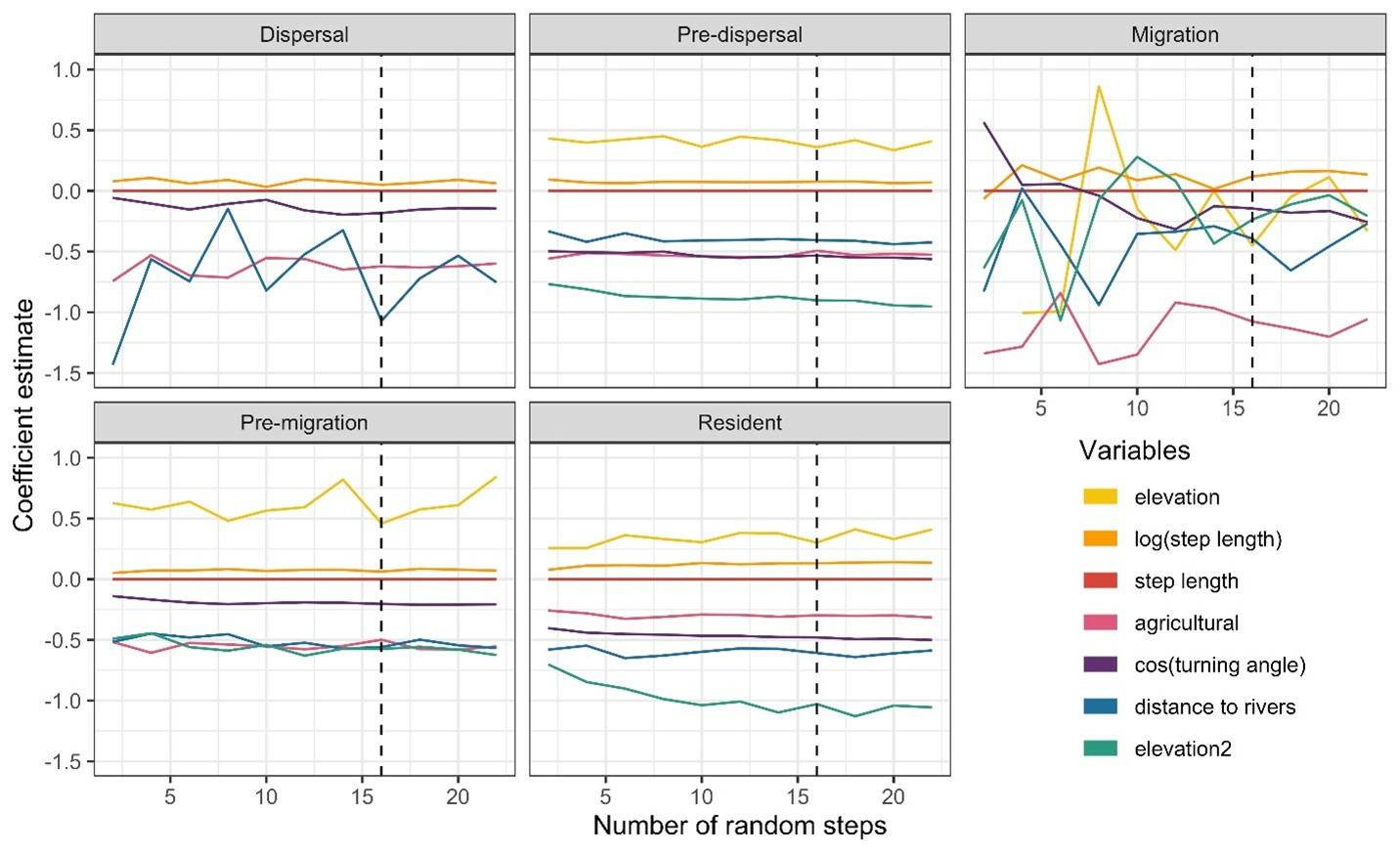

Supplement: S6 Fig — The black dashed lined represents the number of random steps used in the final analyses (16 steps). Estimates for all covariates during migration were very sensitive to the number of steps included. Estimates for elevation during pre-migration were sensitive the number of steps included. Estimates for distance to rivers during dispersal were also sensitive to the number of steps included. Analyses were conducted on subadult white-tailed deer dispersed in southeastern Minnesota, USA, from 2018 to 2021. (TIF) [file pone.0325656.s013.tif]

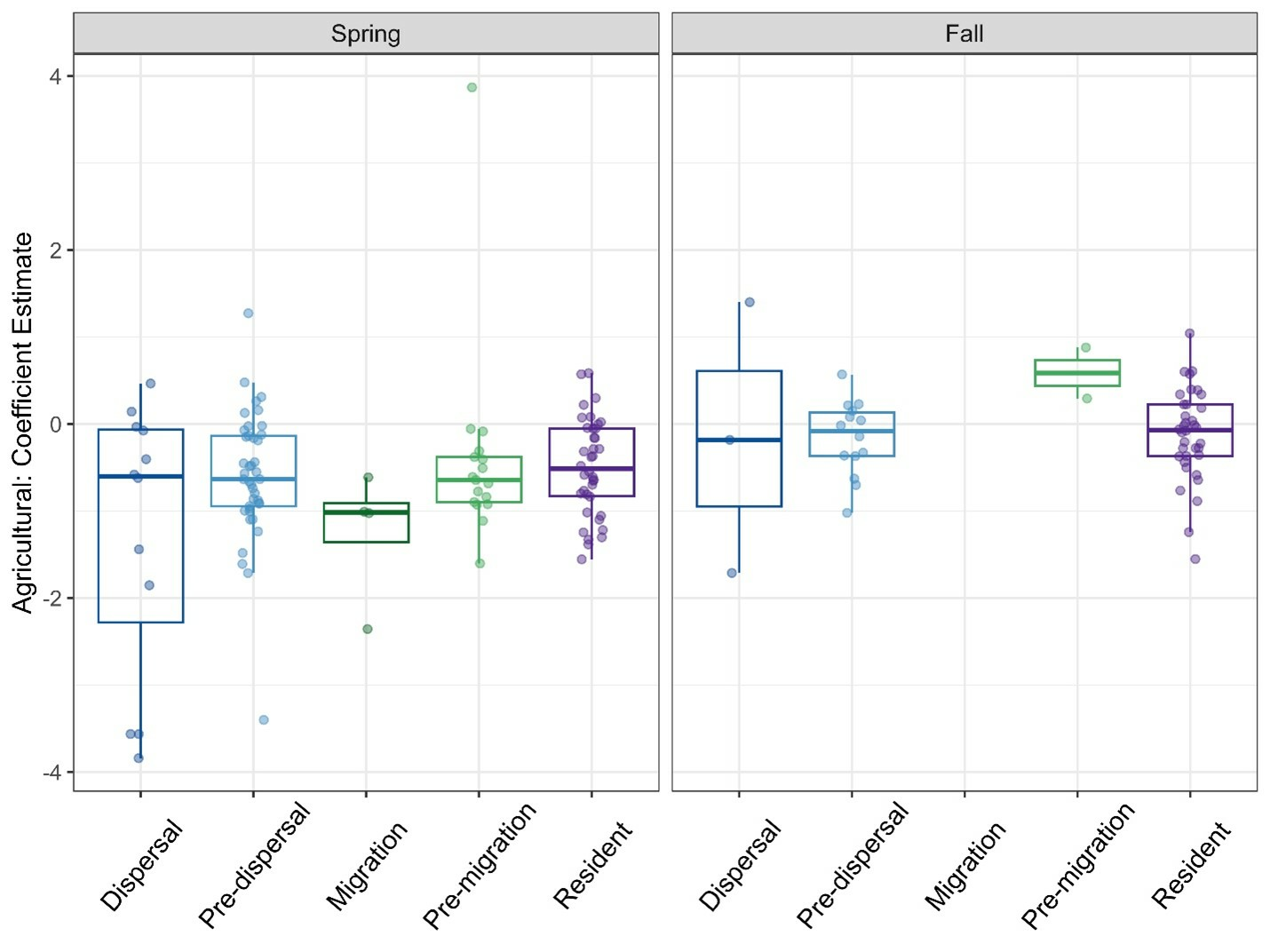

Supplement: S7 Fig — Box and whisker plots represent the full range of data, with the upper and lower quantile defined by the box, and the median value by the middle line. Animals avoided agriculture across all five movement classes in spring. Sample size was limited in autumn for all classes expect pre-dispersal and resident, and should be interpreted with caution. (TIF) [file pone.0325656.s014.tif]
